# Accuracy quantification of a deformable image registration tool applied in a clinical setting

**DOI:** 10.1120/jacmp.v15i1.4564

**Published:** 2014-01-06

**Authors:** Christoph Hoffmann, Sonja Krause, Eva Maria Stoiber, Angela Mohr, Stefan Rieken, Oliver Schramm, Jürgen Debus, Florian Sterzing, Rolf Bendl, Kristina Giske

**Affiliations:** ^1^ Department of Radiation Oncology University Hospital Heidelberg Heidelberg Germany; ^2^ Department of Medical Physics in Radiation Oncology German Cancer Research Center (DKFZ) Heidelberg Germany; ^3^ Medical Informatics Heilbronn University Heilbronn Germany

**Keywords:** deformable image registration, expert‐defined landmarks, target registration error, interobserver variations, adaptive radiation therapy

## Abstract

The purpose of this study was to test the accuracy of a commercially available deformable image registration tool in a clinical situation. In addition, to demonstrate a method to evaluate the resulting transformation of such a tool to a reference defined by multiple experts. For 16 patients (seven head and neck, four thoracic, five abdominal), 30‐50 anatomical landmarks were defined on recognizable spots of a planning CT and a corresponding fraction CT. A commercially available deformable image registration tool, Velocity AI, was used to align all fraction CTs with the respective planning CTs. The registration accuracy was quantified by means of the target registration error in respect to expert‐defined landmarks, considering the interobserver variation of five observers. The interobserver uncertainty of the landmark definition in our data sets is found to be 1.2±1.1mm. In general the deformable image registration tool decreases the extent of observable misalignments from 4‐8 mm to 1‐4 mm for nearly 50% of the landmarks (to 77% in sum). Only small differences are observed in the alignment quality of scans with different tumor location. Smallest residual deviations were achieved in scans of the head and neck region (79%,≤4mm) and the thoracic cases (79%,≤4mm), followed by the abdominal cases (59%,≤4mm). No difference is observed in the alignment quality of different tissue types (bony vs. soft tissue). The investigated commercially available deformable image registration tool is capable of reducing a mean target registration error to a level that is clinically acceptable for the evaluation of retreatment plans and replanning in case of gross tumor change during treatment. Yet, since the alignment quality needs to be improved further, the individual result of the deformable image registration tool has still to be judged by the physician prior to application.

PACS numbers: 87.57.nj, 87.57.N‐, 87.55.‐x

## I. INTRODUCTION

With recent progress in multimodal cancer treatment, radiation oncologists are faced more frequently with long‐term surviving patients with local tumor recurrences that require retreatment. Highly conformal radiation techniques, such as intensity‐modulated radiotherapy that can establish sharp dose gradients, allow the safe retreatment of many tumors.^1^, ^2^, ^3^ However, exact information on the spatial distribution of the previously applied dose is essential for an effective and safe radiotherapy of the recurrence. As patient geometry can deform considerably over time, especially if the patient had surgery, the correlation of old dose distributions in respect to new planning scans can be quite challenging.

Anatomical changes do not only occur between primary treatment and retreatment but also between single fractions of the treatment. For example, MRI studies of uterine and cervical movement have shown large misplacements in the range of several centimeters, especially in the fundus.^4^, ^5^ Patients with cancer of the head and neck tend to lose weight during the treatment course; a consecutive shrinkage of the gross tumor volume of up to 3.9% per treatment day has been demonstrated.^(6)^ For lung cancer, a large mobility of intrapulmonary lesions has been shown.^(7)^

Since correlation with rigid registration methods is insufficient to align deformed anatomy.^8^, ^9^, ^10^ deformable image registration (DIR) methods are required. Software tools that allow DIR of two uncorrelated CT scans might offer assistance for adaptive radiation strategies and retreatment planning.^11^, ^12^ Yet, data on the applicability and reliability in daily clinical routine are scarce.

In this study, we present a possible approach for analyzing the accuracy of a commercially available DIR tool. Such tools claim the reliability of the established transformations between different CT scans without reporting the extent of the respective uncertainties in detail. However, the transformation accuracy will strongly depend on the clinical data used for registration, and therefore determine the applicability of these tools in daily clinical routine. Since proprietary products rarely allow the evaluation of the registration quality for the existing data sets, clinicians cannot decide whether the accuracy of the obtained transformations is sufficient for the specific applications. To overcome this obstacle, we demonstrate a method to put an arbitrary DIR tool to the test. Using a commercially available tool, we additionally present the results we have gained by applying this specific tool to our datasets.

## II. MATERIALS AND METHODS

### A. Patient data

Sixteen patients were selected for this investigation. Tumor sites were head and neck (seven patients), thoracic (four) and abdominal (five). Within these groups patient datasets were selected consecutively. Each patient had obtained a planning CT scan with 2 mm slice thickness for thoracic and abdominal cases and 3 mm slice thickness for head and neck cases. All datasets were complemented by regular image guidance. The fraction CTs were acquired on an in‐room kV‐CT scanner PRIMATOM (Siemens Medical Solutions, Malvern, PA). Scans for thoracic patients were obtained with a breath‐hold technique.

**Figure 1:**
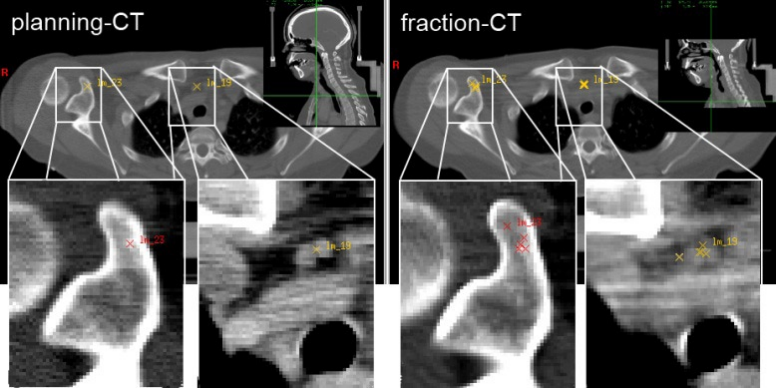
Expert‐defined landmarks in a head and neck patient: (left) planning CT with two defined landmarks located in a bony structure (red cross) and next to a vascular system (yellow cross); (right) corresponding position of the same landmarks in the fraction CT independently identified by five observers. Note: To illustrate the interobserver variation in 2D, all relocated landmark positions were projected onto the same transversal slice, neglecting their real slice position.

For the DIR, the planning CT and a fraction CT (obtained approximately halfway through the treatment course) were chosen. For every patient, 30‐50 landmarks were set by an expert on recognizable spots on the planning CT. Clearly identifiable patient‐specific structures (e.g., fissures in bony structures) were included to maximize the number of landmarks. Structures believed to be prone to movement, such as calcifications in soft tissue or bifurcations of small vessels, as well as structures believed to be more static, such as bony protrusions, were marked. The same landmarks, defined on the planning CTs, were set independently by five observers (four radiation oncologists and one medical physicist) on the respective fraction CTs. Two exemplary landmarks are displayed in Fig. 1. All landmarks were additionally classified by an expert according to the expected deformability of its surrounding tissue (e.g., calcifications were frequently located in blood vessels embedded in soft tissue and were classified as “soft tissue”).

### B. Commercial software under investigation

The DIR was performed with Velocity AI Version 2.6.1 (Velocity Medical Systems, Atlanta, GA). This tool is distributed as a stand‐alone product for radiation oncology, which enables the user to compare different DICOM conformal datasets of different image modalities including their visualization, registration, and fusion. Velocity AI offers a rigid and a deformable image registration method. The deformable image registration is mutual‐information‐based and models the deformation using a modified B‐spline approach.^(13)^

### C. Registration procedure

The evaluation of the registration quality is performed using the comparison of the resulting alignment in respect to expert‐defined landmarks by means of the target registration error (TRE)^(14)^ (also referred as distance to agreement^(15)^). Since the calculated transformation cannot be extracted directly, we used the following scheme. The expert‐defined landmarks were transferred to volumetric images, which were artificially generated by marking the voxels containing the respective landmarks (referred to as fraction landmark CTs). That image stack can be deformed by applying the same transformation as calculated for the fraction CT. After the optimal transformation, the marked voxels should have been moved to the position of the landmarks defined on the planning CT. Any difference describes a residual deviation due to insufficient transformation.

**Figure 2:**
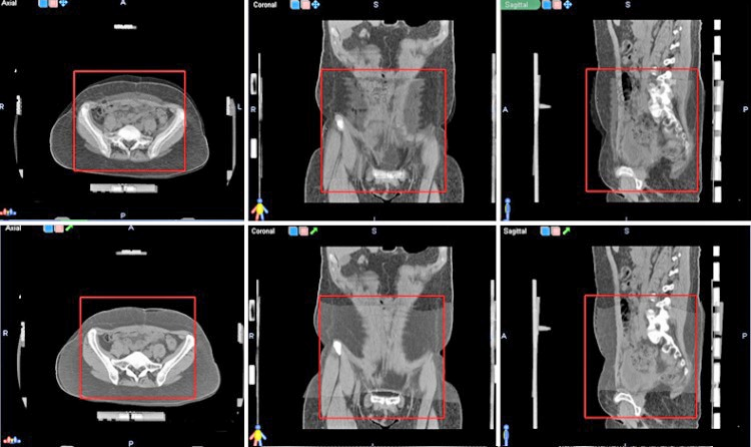
Definition of the region of interest (ROI) for DIR in Velocity AI: (first row) the planning CT and the original fraction CT are superimposed; (second row) a fusion of the planning CT and the deformed fraction CT. Only image information inside the red ROI is used to determine the transformation.

It should be noted that hereby the positioning accuracy of the landmark was reduced to the voxel size. All three data sets — the planning CT, the kV fraction CT, and the corresponding fraction landmark CT — were imported to Velocity AI. The planning CT was loaded as the primary volume. It served as reference for all of the following volumes. The fraction CT was loaded as the secondary volume. Both nondeformed scans are shown in fusion mode in Fig. 2.

For this study, a region of interest (ROI) was defined in the primary volume to exclude any external positioning and fixation devices in order to avoid their influence in the registration process. A typical ROI used in this study is displayed in Fig. 2. Attention was paid to include all relevant anatomical structures including those where landmarks were defined. For all scan pairs the auto registration type “rigid and deformable multipass” was chosen (“deformable grid settings” and “contrast option” on default). By this selection, first the fraction CT was rigidly registered in respect to the planning CT. Subsequently, the DIR was accomplished within the ROI by warping sections of the secondary volume to fit the chosen region. Since the tool does not offer an alternative to evaluate the calculated vector field, expert‐defined landmarks were used to assess the calculated transformation. Therefore, an artificial CT derived from the fraction CT was created containing the landmarks only (fraction landmark CT).

To apply the calculated transformation to the fraction landmark CT, a correlation to the coordinate system of the fraction CT was required. Therefore, both scans must refer to the same DICOM frame of reference, which was achieved by the auto registration option “DICOM”. The previously gained deformation between the planning CT and the fraction CT can then be applied to the fraction landmark CT. The generated deformed fraction landmark CT was exported to the file system for comparison with the unchanged planning landmark CT using an externally developed evaluation tool.

### D. Evaluation procedure

The evaluation was performed using VIRTUOS, an in‐house build treatment planning system, which serves as a research and development platform at DKFZ.^(16)^ The registration accuracy is reported using the 3D Euclidean distances between the corresponding landmarks in each CT pair to be registered. These distances are called TREs when referring to landmark distances after their transformation resulting from a registration process. For easier handling, we will use the term TREs also for distances prior to registration, indicating the difference where appropriate. The initial distribution of the TREs, prior to registration, is reported using the mean values of each landmark accompanied by the standard deviations of the five observer‐definitions. Since few landmark definitions showed outlier characteristics, outlier detection with a six‐sigma criterion in regard to the mean of the narrowest distribution of five‐minus‐one observers was performed. After the transformation of the fraction landmark CT, the resulting TREs were detected.

The distributions of the distances prior and after the registration were analyzed for all landmarks. In addition, all classified in different tumor location groups, as well as in different landmark features groups. The landmark features differed depending on whether the landmark was embedded in bony anatomy or in soft tissue regions.

## III. RESULTS

### A. Interobserver variations

A single landmark is characterized as the average of the different observers. The deviation of the landmark positions around the average reflects the interobserver variation and ranged between 0 and 17.8 mm. The resulting distribution of the standard deviations has a modal value of 1.2 mm, with the quartiles of 0.6 mm (25%), 0.9 mm (50%), and 1.3 mm (75%).

### B. Range of residual misalignments: best and worst case

**Figure 3:**
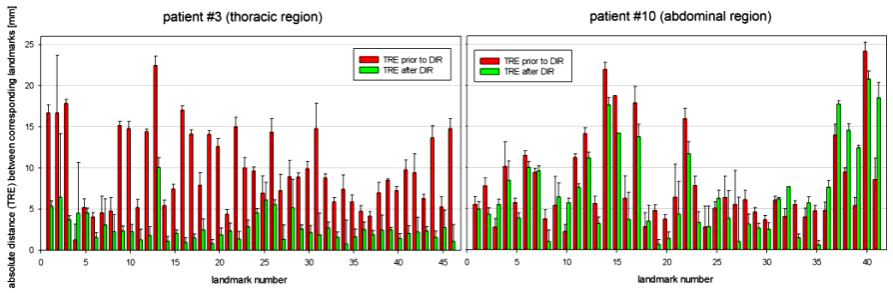
3D distances of landmarks between the planning CT and the respective fraction CT for the two patients with best and worse achieved registration quality; distances prior to the DIR (red bars) and the target registration error (green bars) after applying the DIR. The error bars indicate the interobserver uncertainty for each landmark.

Figure 3 shows the averaged TREs for all landmarks for the best case and worst case results. The red bars indicate the determined distances prior to DIR. The corresponding remaining distances after the registration are plotted with green bars. The error bars indicate the interobserver uncertainty of each landmark. The left panel in Fig. 3 shows all TREs of the scan pair that showed the best registration result. The initial TREs (red bars) were in the range between 5‐22.5 mm. After DIR, the range of the TREs was decreased to 1‐10 mm. The right panel displays the TREs of all landmarks of the scan pair showing the worst registration result out of the analyzed patient cohort. The initial TREs were in the range of 4‐24 mm. The deviations after the DIR did not show any decrease, considering the 1‐sigma error bars. In some cases the mean deviations were even slightly increased, indicating a bigger misalignment between anatomical structures.

### C. Residual misalignments: patient cohort

**Figure 4:**
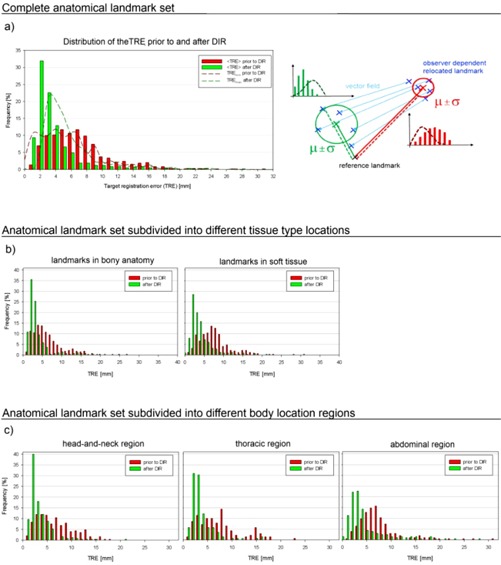
The bars represent the distribution of the distances prior to (red) and after (green) registration. (a) TRE distribution for all landmarks. The better the registration quality, the narrower and more shifted towards zero the distribution should be. The dashed lines indicate the uncertainty based on interobserver variations. The sketch on the right visualizes the values sampled in these distributions. The continuous red (green) lines are considered for the distribution plotted using bars and the dashed lines indicate distances sampled for the dashed distributions; μ indicates the mean landmark position and σ is the standard deviation between the five positions of the different observers. (b) Same illustration when landmarks are subdivided in two groups depending on their tissue surroundings, in bony anatomy (left) or in soft tissue (right). (c) Subdivision of all landmarks into three groups depending on the investigated tumor location: head and neck (left), thoracic (center), and abdominal regions (right).

Figure 4(a) shows the distribution of the TREs using the anatomical landmarks of all patients. In general prior to image registration, the landmark deviations show a broader distribution (red), compared to the ones after image registration (green). Additionally, the green distribution shows a clear shift to smaller distances. Initially, 28.5% of the landmarks show a TRE of 4 mm or less. After applying DIR, the number of landmarks with a TRE of 4 mm or less increases to 77% with a maximum of 32% at 2 mm. According to that, 48.5% of the landmarks are shifted from the initial range of 4‐8 mm to the range of 1‐4 mm. The dashed lines represent the respective distributions considering the interobserver uncertainty when locating the landmarks. The interobserver variation allows for an uncertainty estimation of the resulting distributions. The red dashed line indicates the distribution of the TRE of all defined landmarks, in cases where the mean landmark distance overestimates the real one. These distances are calculated with respect to the mean distance minus one standard deviation of the five expert‐defined positions. The green dashed line shows the distribution of the remaining TREs in situations where the mean distances underestimate the real ones. These frequencies are calculated in respect to the mean distance plus one standard deviation.

### D. Influence of the landmark surroundings

All landmarks are classified as to whether they are located in bony anatomy or in soft tissue to evaluate the influence of the tissue type of its surroundings on the quality of the DIR. Figure 4(b) shows the corresponding TRE distributions. Both types of landmarks show comparable improvement after the transformation derived by the DIR.

Figure 4(c) shows the results of the image registration subdivided according to the tumor location. Slightly better alignment was achieved in scans of the head and neck anatomy (79%,≤4mm) and the thoracic cases (79%,≤4mm), followed by abdominal cases (59%,≤4mm).

## IV. DISCUSSION

We have shown that the DIR implemented in Velocity AI is feasible to improve mean target registration errors to 4 mm or less with about half of the landmarks in the investigated clinical data. It has to be kept in mind that our dataset was acquired at up to 3 mm slice thickness, and the registration and evaluation quality is also dependent on the scan resolution. The image registration quality was found to be independent of whether a landmark was located in rigid structures (bones or vicinity of bones) or in soft tissue. Comparing the registration quality of scans with different anatomical regions, we see a similar reduction of the TREs in all cases. However, slightly better results are observed in head and neck and thoracic cases, followed by abdominal cases. One possible reason for the inferior quality of the resulting transformation accuracy in the abdominal region could be the lower contrast or its sensitivity to organ deformations (e.g., caused by varying filling conditions). The slightly superior registration quality in the head and neck anatomy may be explained by the higher tissue contrast and more rigid anatomical components.

The DIR accuracy of the used software tool, Velocity AI, was also assessed in alternative studies using thoracic patient datasets for lung tissue alignment^(17)^ and abdominal patient datasets, as well as a phantom dataset.^(18)^ Both studies report and conclude a comparable good registration quality applying Velocity AI. The quantified lung alignment accuracy of (3.1±0.8)mm to (8.0±3.1)mm ^(17)^ is also assessed in regard to expert‐defined landmarks. Also the assessment of the alignment quality of the abdominal cases showed good results with only a small fraction of misalignments up to 15 mm in clinical datasets.^(18)^ Of course, the registration quality depends on the quality of the used datasets, and the number and positions of the reference landmarks.

To establish a comparison for evaluation of the DIR, expert‐defined anatomical landmarks are frequently used.^19^, ^20^ Despite of the known drawbacks of this method, no superior and pragmatic approach has been established so far. Nevertheless, the validity of this approach needs to be considered when such results are presented and discussed. Particularly, when generalizations are made to summarize results and draw conclusions in a concise fashion, it should be kept in mind that all derived data are based on two assumptions: firstly, that the expert is correct, and secondly, that whatever is true for only few identifiable landmarks is also true for the remaining anatomy, even if the intrinsically high‐contrasted landmarks were not present. Interobserver variation is an important factor whenever expert‐defined references are used. The assessment of interobserver variations can help to identify better treatment options in radiation therapy.^(21)^ In particular, the assessment of image registration quality needs to account for interobserver variations.^(15)^ Still, the expert‐defined landmarks, especially if interobserver variations are considered, allow quantification to an extent that can communicate the registration quality beyond the visual impression of two fused image scans presented by most available products. So, we consider a purely visual inspection of two fused image modalities insufficient to decide the applicability of the result to a specific objective. Products offering DIR should also implement a functionality to evaluate each single registration result with regard to the specific application (e.g., allowing for landmark definitions or volume based analysis)^22^, ^23^ and assisting in the evaluation process for each single patient if necessary.

Although it could be shown that the target registration errors for approximately 50% of the landmarks were reduced beneath 4 mm, there is still the question of whether this is sufficient for application in clinical routine. While previous DIR alignment studies reported subvoxel accuracy,^24^, ^25^ we observed in our datasets still about half of all defined landmarks showing a bigger residual misalignment than 4 mm after registration (diagonal voxel size∼3mm). From the current clinical point of view, an error of 2‐4 mm of the resulting transformation is sufficient for the evaluation of retreatment plans and replanning in case of gross change of tumor extensions during treatment, as the error is comparable to the observed extent of interfractional deviations of most anatomical sites. For example, in the case of the uterine cervix, the mean displacement of the posterior cervix taken on two consecutive days was 4.1 mm superior/inferior, 2.7 mm anterior/posterior, and 0.3 mm laterally.^(4)^ A study on ten patients treated for head and neck cancer showed a mean displacement of the CTV including the lymph nodes of 1.8 mm over the course of treatment.^(6)^

However, in some clinical situations, an accuracy of 2‐4 mm might not be enough. For example, the retreatment of tumor close to the optic nerve or the chiasm might require a higher precision to ensure the preservation of eyesight. In either case, the clinician's task is to critically scrutinize the results of automatic DIR tools.

Due to technical innovations the complexity of radiotherapy treatment of a broad range of tumor entities is increasing. Therefore, sophisticated tools to support the clinicians are required. A tool for reliable dose summation in deforming anatomy would be of benefit for the evaluation of retreatment plans. The effect of a volume change on the accumulated dose, however, has not been fully investigated yet^(26)^ and is beyond the scope of this study.

With the advent of more sophisticated automatic tools in contouring and planning, the clinician's challenge is to critically evaluate the accuracy of the respective tools. The method presented in this study can be an approach to assess the accuracy of DIR tools. However, in the end, clinical experience must have the last word.

## V. CONCLUSIONS

The investigated commercially available DIR software is capable of reducing a mean residual misalignment of anatomical structures considerably for our datasets. For the evaluation of retreatment plans and replanning in case of gross tumor changes, its accuracy is in general clinically acceptable. However, suitability for all cases, especially for more complex ones, needs still to be improved further. The individual result of the DIR has still to be judged by the clinician. Therefore, development of tools to analyze DIR accuracy is essential for the clinical application of DIR methods.
